# Elevated ADAMTS13 Activity is Associated with Poor Postoperative Outcome in Patients Undergoing Liver Resection

**DOI:** 10.1038/s41598-018-34794-w

**Published:** 2018-11-14

**Authors:** Stefanie Haegele, Jennifer Fuxsteiner, David Pereyra, Christoph Koeditz, Benedikt Rumpf, Clara Schuetz, Christian Schwarz, Christine Brostjan, Thomas Gruenberger, Patrick Starlinger

**Affiliations:** 1 Department of Surgery, Medical University of Vienna, General Hospital of Vienna, Vienna, Austria; 2 Department of Surgery, Kaiser Franz Josef Hospital, Vienna, Austria

## Abstract

Recently, von-Willebrand-Factor (vWF) has been shown to correlate with postoperative liver dysfunction (LD). Accordingly, “disintegrin-like metalloprotease with thrombospondin type1 motif” (ADAMTS13) is known to cleave vWF in less active fragments. Thus, we aimed to evaluate the diagnostic potential of ADAMTS13-activity (ADAMTS13-AC) to identify patients with postoperative LD after hepatectomy. Accordingly 37 patients undergoing hepatectomy for different neoplastic entities were included in this study. Plasma ADAMTS13-AC and vWF-Ag were measured 1 day prior to (preOP), 1 and 5 days (POD1/5) after hepatectomy. In accordance to the ISGLS-criteria LD was prospectively recorded. In this context, perioperative ADAMTS13-AC- and vWF-Ag/ADAMTS13-AC-ratio- levels revealed a significant increase after hepatectomy. Accordingly, elevated vWF-Ag/ADAMTS13-AC-ratio significantly predicted LD (preOP AUC: 0.75, p = 0.02; POD1 AUC: 0.80, p = 0.03). Patients who fulfilled our perioperative vWF-Ag/ADAMTS13-AC-ratio cut-off-levels (preOP: ≥116, POD1: ≥165) suffered from significantly higher incidences of LD (preOP: 70% vs. 30%, p = 0.01; POD1: 83% vs. 17%, p = 0.001). In conclusion, perioperative ADAMTS13-AC measurement may serve as a useful parameter to early detect high-risk patients developing postoperative LD prior to liver resection in patients suffering from hepatic malignancies. Indeed, further investigations have to be performed to consolidate its role as a predictive marker for LD.

## Introduction

A sufficient ability of the liver to recover after its resection is the most significant factor determining postoperative outcome after hepatectomy. If hepatic regeneration is impaired, postoperative liver dysfunction (LD) occurs and thereby markedly increases the risk of postoperative morbidity and mortality^1,2^. Therefore, preoperative risk assessment to predict postoperative LD and clinical outcome is of major importance.

With respect to this, hepatic microcirculation is known to play a crucial role in the complex process of liver regeneration (LR). It has been hypothesized that one of the most important trigger mechanism of initiating the regeneration process after liver resection is the relative increase in sinusoidal blood flow after loss of liver mass^3^. If the liver is reduced in its size the whole amount of hepatic venous blood has to run through the reduced remaining liver lobe. This relative increase in blood flow causes a concomitant increase of intrahepatic shear forces and induces a stimulus on the endothelial surface, which in turn triggers a signaling cascade, essential for hepatic regeneration^3–5^. Accordingly, von Willebrand factor (vWF) is secreted upon endothelial cell activation and is known to form so called vWF–strings under increased shear conditions. These complexes, formed through ultra large vWF molecules, are essential for localized platelet aggregation and activation^6,7^. In this context it should be noted that platelets and their different granule contents have recently been shown to play a pivotal role for the improvement of the regenerative potential after liver resection^8–10^. Importantly, we provided evidence that an immediate vWF increase after induction of LR has a direct effect on site specific platelet accumulation and postoperative liver function recovery^11^.

However, increased accumulation or insufficient degradation of vWF–strings can cause thrombotic events and may led to a worsened outcome after partial hepatectomy^12,13^. Accordingly, vWF is proteolysed via the so called disintegrin-like and metalloprotease domain with thrombospondin type 1 motif (ADAMTS13). ADAMTS13 is exclusively produced in the liver, secreted as an active enzyme and therefore steadily active in plasma. It cleaves vWF-strings at the A2 domain, which is folded under normal flow conditions. Increased shear forces, as observed after liver resection, have been shown to force the unfolding of the ADAMTS13-cleaving site and proteolysis of vWF–strings^7,14^. Indeed, several studies have addressed the role of ADAMTS13 in liver disease. Still, little is known about the role of ADAMTS13 and its impact on postoperative complications after liver resection in humans^13,15,16^.

In this context, Thrombospondin 1 (TSP-1), a homotrimeric glycoprotein with well-known functions in haemostasis and angiogenesis was found to bind to the A2 domain of vWF, leading to a competition with ADAMTS13 and therefore protecting subendothelial vWF from being cleaved by circulating ADAMTS13^17^. TSP-1 can be produced by several different cell types but its major source is represented by platelets and endothelial cells^17,18^. In plasma, it circulates only in very low concentrations however, after platelet or endothelial cell activation it can be released leading to an upregulation of the plasma TSP-1 levels up to 100 times of the initial concentration^19^. Accordingly, in the past TSP-1 was shown to represent a negative regulator of LR by activation of latent transforming growth factor β1 and recently we were able to show that high TSP-1 levels significantly predicted poor postoperative outcome after liver resection in humans^20,21^.

Thus, we here aimed to evaluate the role of ADAMTS13-activity (ADAMTS13-AC) and its potential as a predictive marker for risk assessment in human LR after partial hepatectomy via the evaluation of perioperative blood dynamics.

## Material and Methods

### Patients and study cohort

A total of 37 consecutive patients, undergoing partial hepatectomy between February 2012 and March 2014, at the general hospital of Vienna, were included in this study. Patients with different neoplastic entities were evaluated, namely metastatic colorectal cancer (mCRC, N = 14), hepatocellular carcinoma (HCC, N = 14) and cholangiocellular carcinoma (CCC, N = 9). Patient’s plasma ADAMTS13-AC- as well as vWF-Ag- levels and routine laboratory parameters were evaluated one day prior to surgery (preOP) and one day (POD1) as well as 5 days (POD5) after liver resection. In addition, specific perioperative characteristics of all patients were prospectively recorded and are listed in Table 1 and Table 2. The extent of resection was classified according to the “IHPBA Brisbane 2000 nomenclature” (<3 segments = minor, >3 segments = major). Furthermore, this study involves the analysis of ADAMTS13-AC as well as patient data comparison and was approved by the institutional ethics committee of the “Medical University of Vienna”, in accordance with the “Declaration of Helsinki” (59^th^ WMA General Assembly, Seoul, Republic of Korea, October 2008). All patients gave written informed consent. Furthermore, the trial was registered at a clinical trials registry (ClinicalTrilas.gov Identifier NCT02113059).

**Table 1:** PreOP levels (LD & vWF-Ag/ADAMTS13-AC ratio), patient demographics, outcome- and laboratory parameters.

Parameter	PreOP (N = 37)	LD (N = 37)		P - value	vWF-Ag/ADAMTS13-AC ratio (N = 37)		P - value
		No (N = 27)	Yes (N = 10)		<116 (N = 24)	≥116 (N = 13)	
	N = /Median (Range/%)				N = /Median (Range/%)		
**Age (years)**	65.6 (40.0–86.1)	65.6 (40.0–86.1)	67.5 (51.1–81.8)	0.80	64.3 (40–86.0)	69.0 (55.1–86.1)	0.56
**Sex**							
Male	30 (81.1)	22 (81.5)	8 (80.0)	0.92	22 (91.7)	8 (61.5)	**0**.**03**
Female	7 (18.9)	5 (18.5)	2 (20.0)		2 (8.3)	5 (38.5)	
**Neoplastic entity**							
mCRC	14 (37.8)	11 (40.7)	3 (30.0)	0.81	12 (50.0)	2 (15.4)	0.06
HCC	14 (37.8)	10 (37.0)	4 (40.0)		6 (25.0)	8 (61.5)	
CCC	9 (24.4)	6 (22.3)	3 (30.0)		6 (25.0)	3 (23.1)	
**Hepatic resection**							
Major	24 (64.9)	15 (55.6)	9 (10.0)	0.05	14 (58.3)	10 (76.9)	0.26
Minor	13 (35.1)	12 (44.4)	1 (90.0)		10 (41.7)	3 (23.1)	
**Cirrhosis**							
yes	11 (29.7)	7 (18.9)	4 (40.0)	0.41	4 (16.7)	7 (53.8)	**0**.**02**
no	26 (70.3)	20 (74.1)	6 (60.0)		20 (83.3)	6 (46.2)	
**RBC-transfusion (intraoperative)**							
yes	5 (100.0)	3 (60.0)	2 (40.0)	0.48	1 (20.0)	4 (80.0)	**0**.**02**
**LD**							
yes	10 (100.0)				3 (30.0)	7 (70.0)	**0**.**01**
**90-days mortality**							
**yes**	**1 (100.0)**	**0 (0.0)**	**1 (100.0)**	**0.09**	**0 (0.0)**	**1 (100.0)**	**0.17**
**Preoperative Parameters**							
ADAMTS13-AC IU/ml	0.6 (0.4–1.1)	0.6 (0.4–1.1)	0.7 (0.5–1.0)	**0**.**04**	0.6 (0.4–0.8)	0.7 (0.5–1.1)	**0**.**001**
vWF-Ag %	165.0 (55.8–420.0)	155.0 (88.5–253.0)	184.5 (55.8–420.0)	**0**.**048**	139.5 (55.8–214.6)	185.0 (160.0–420.0)	**0**.**001**
vWF-Ag/ADAMTS13-AC ratio	91.5 (42.0–301.1)	84.8 (42.0–193.3)	127.3 (42.4–301.1)	**0**.**02**			
TSP-1 ng/ml	39.0 (17.2–195.9)	41.7 (17.2–179.4)	37.0 (23.5–195.9)	0.78	40.3 (17.3–179.4)	38.1 (17.2–195.9)	0.84
SB mg/dl	0.7 (0.2–2.3)	0.7 (0.2–2.3)	1.1 (0.5–2.0)	0.15	0.7 (0.2–2.1)	0.6 (0.3–2.3)	0.67
PT%^a^	96.0 (40.0–150.0)	98.0 (61.0–150.0)	80.0 (40.0–120.0)	0.13	98.5 (61.0–150.0)	83.0 (40.0–124.0)	0.11
ALP U/l	85 (45–300)	85 (45–230)	93 (64–300)	0.27	84 (45–196)	105 (61–300)	**0**.**03**
GGT U/l	71.5 (11.0–710.0)	68.5 (11.0–710.0)	112.0 (31.0–699.0)	0.20	65.0 (11.0–299.0)	110.0 (51.0–710.0)	**0**.**01**
AST U/l	29.5 (17.0–208.0)	27.5 (17.0–75.0)	34.0 (21.0–208.0)	0.17	27.0 (17.0–62.0)	35.0 (21.0–208.0)	**0**.**03**
ALT U/l	33.0 (7.0–112.0)	29.0 (7.0–112.0)	39.5 (21.0–83.0)	0.16	27.0 (7.0–112.0)	39.0 (9.0–83.0)	0.45
Albumin g/l	41.9 (31.5–48.5)	42.7 (35.5–48.5)	40.1 (31.5–47.2)	0.06	42.6 (35.5–48.5)	40.7 (31.5–45.7)	0.10
Platelets (x10^3/µl)	211.0 (86.0–492.0)	210.0 (86.0–492.0)	217.5 (152.0–284.0)	0.96	210.0 (92.0–313.0)	224.0 (86.0–492.0)	0.86

ADAMTS13, disintegrin-like and metalloproteinase with a thrombospondin type 1 motif member 13; ALP, alkaline phosphatase; ALT, alanine amino transferase; AST, aspartate amino transferase; CCC, cholangiocellular carcinoma; CTx, chemo therapy; GGT, gamma glutamyl transferase; HCC, hepatocellular carcinoma; LD, liver dysfunction; mCRC, metastatic colorectal cancer; PreOP, preoperative; PT, prothrombin time; RBC-transfusion, red blood cell transfusion; SB, serum bilirubin; TSP-1, thrombospondin 1; vWF-Ag, von Willebrand-Factor antigen.

^a^PT is expressed in relation to the coagulation time of a healthy person (i.e. Quick). Accordingly, it is illustrated in percentage.

**Table 2:** POD1 levels (LD & vWF-Ag/ADAMTS13-AC ratio), patient demographics, outcome- and laboratory parameters.

Parameter	POD1 (N = 37)	LD (N = 37)		P - value	vWF-Ag/ADAMTS13-AC ratio (N = 28) *9 cases are missing*		P - value
		No (N = 27)	Yes (N = 10)		<165 (N = 21)	≥165 (N = 7)	
	N = /Median (Range/%)				N = /Median (Range/%)		
**Age (years)**	65.6 (40.0–86.1)	65.6 (40.0–86.1)	67.5 (51.1–81.8)	0.80			
**Sex**							
Male	30 (81.1)	22 (81.5)	8 (80.0)	0.92	17 (81.0)	1 (14.3)	0.78
Female	7 (18.9)	5 (18.5)	2 (20.0)		4 (19.0)	6 (85.7)	
**Neoplastic entity**							
mCRC	14 (37.8)	11 (40.7)	3 (30.0)	0.81	11 (52.4)	2 (28.6)	0.26
HCC	14 (37.8)	10 (37.0)	4 (40.0)		3 (14.3)	3 (42.8)	
CCC	9 (24.4)	6 (22.3)	3 (30.0)		7 (33.3)	2 (28.6)	
**Hepatic resection**							
Major	24 (64.9)	15 (55.6)	9 (10.0)	0.05	13 (61.9)	6 (85.7)	0.24
Minor	13 (35.1)	12 (44.4)	1 (90.0)		8 (38.1)	1 (14.3)	
**Cirrhosis**							
yes	11 (29.7)	7 (18.9)	4 (40.0)	0.41	3 (14.3)	2 (28.6)	0.39
no	26 (70.3)	20 (74.1)	6 (60.0)		18 (85.7)	5 (71.4)	
**RBC-transfusion (postoperative)**							
yes	4 (100.0)	0 (0.0)	4 (100.0)	**0**.**001**	0 (0.0)	3 (100.0)	**0**.**001**
**LD**							
yes	10 (100.0)				1 (16.7)	5 (83.3)	**0**.**001**
**90-days mortality**							
yes	1 (100.0)	0 (0.0)	1 (100.0)	0.09	0 (0.0)	1 (100.0)	0.09
**Postoperative Parameters**							
ADAMTS13-AC IU/ml	0.5 (0.3–0.7)	0.5 (0.3–0.7)	0.6 (0.5–0.7)	**0**.**02**	0.5 (0.3–0.7)	0.6 (0.5–0.7)	**0**.**004**
vWF-Ag %	280.0 (49.4–445.0)	256.0 (128.3–422.0)	350.2 (49.4–445.0)	**0**.**02**	241.0 (49.4–343.5)	384.3 (243.5–445.0)	**0**.**001**
vWF-Ag/ADAMTS13-AC ratio	132.7 (27.7–280.4)	119.2 (49.7–267.3)	214.0 (27.7–280.4)	**0**.**02**			
TSP-1 ng/ml	62.8 (14.9–303.0)	55.1 (14.9–303.0)	93.6 (51.4–157.4)	**0**.**005**	59.4 (14.9–303.0)	94.1 (55.1–242.0)	**0**.**02**
SB mg/dl	1.5 (0.4–8.2)	1.1 (0.4–3.8)	2.7 (1.2–8.2)	**0**.**001**	1.1 (0.4–3.8)	3.0 (0.6–8.2)	**0**.**03**
PT% ^a^	54 (29–90)	58 (40–90)	46 (29–64)	**0**.**002**	57 (41–90)	53 (36–64)	0.20
ALP U/l	70 (35–591)	59 (35–152)	82 (44–591)	0.08	58 (35–148)	91 (44–591)	**0**.**048**
GGT U/l	65.0 (6.0–462.0)	63.0 (6.0–462.0)	121.5 (41.0–431.0)	**0**.**04**	63.0 (6.0–241.0)	112.0 (38.0–462.0)	0.06
AST U/l	377 (56–3747)	356 (56–1825)	508 (188–3747)	0.10	377 (56–819)	343 (124–3747)	0.84
ALT U/l	347.0 (70.0–2944.0)	295.0 (70.0–1049.0)	473.5 (164.0–2944.0)	0.19	357.0 (70.0–847.0)	338.0 (86.0–2944.0)	0.92
Albumin g/l	29.6 (23.3–37.5)	29.8 (23.3–37.5)	29.5 (24.6–32.4)	0.39	30.8 (23.3–37.5)	28.6 (26.4–32.4)	0.19
Platelets (x10^3/µl)	178.0 (70.0–391.0)	178.0 (70.0–391.0)	185.5 (77.0–316.0)	0.65	178.0 (70.0–391.0)	204.0 (109.0–316.0)	0.19

ADAMTS13, disintegrin-like and metalloproteinase with a thrombospondin type 1 motif member 13; ALP, alkaline phosphatase; ALT, alanine amino transferase; AST, aspartate amino transferase; CCC, cholangiocellular carcinoma; CTx, chemo therapy; GGT, gamma glutamyl transferase; HCC, hepatocellular carcinoma; LD, liver dysfunction; mCRC, metastatic colorectal cancer; POD1, postoperative day 1; PT, prothrombin time; RBC transfusion, red blood cell transfusion; SB, serum bilirubin; TSP-1, thrombospondin 1; vWF-Ag, von Willebrand Factor antigen.

^a^PT is expressed in relation to the coagulation time of a healthy person (i.e. Quick). Accordingly, it is illustrated in percentage.

### ADAMTS13-AC Measurement

Perioperative ADAMTS13-AC levels were evaluated via commonly available chromogenic ELISA for the determination of its activity in human plasma (Technozym ADAMTS-13 ACTIVITY Elisa, Technoclone, Vienna, Austria). Briefly, GST-vWF73 substrate were added to anti-GST antibody coated plates and incubated with patient’s plasma samples. Therefore, ADAMTS13 in the patient’s blood samples will then cleave vWF73 substrate. The specific cleavage site was further visualized via addition of HRP conjugate against the cleavage site of vWF substrate and measurement of its density at 450 nm wavelength. Accordingly, the amount of colored substrate is directly related to the extent of patient’s ADAMTS13-AC. The range of ADAMTS13-AC which is to be expected in the normal population varies from 0.40 to 1.30 IU/ml and the assays range of detection varies from 0.002 IU/ml (detection limit) to 1.05 IU/ml.

### VWF-AG Measurement

Perioperative vWF-Ag levels were measured through the local routine laboratory via enzyme linked immunosorbent assay (ELISA [Asserachrom vWF:FVIIIB – Elisa, Stago, Neuffen, Germany]). The assay indicates the capacity of vWF-Ag to bind to the factor VIII. The range of vWF-Ag which is to be expected in the normal population varies from 60–180% and the assays lowest detection limit is about 1%. Accordingly, blood was collected perioperatively into 3 ml 9NC Coagulation sodium citrate 3,2% vacuette tubes and was immediately sent to the local laboratory for further analyzes.

### TSP-1 Measurement

Perioperative TSP-1 levels were analyzed by means of commercially available enzyme-linked immunosorbent assay (Quantikine®; R&D Systems, Minneapolis, Minnesota, USA) for human TSP-1

### Definition and classification of postoperative ld and morbidity

To assess postoperative LD, the definition published by the international study group of liver surgery (ISGLS) were modified as previously described^8^. Accordingly. LD was defined by an abnormal serum bilirubin level and prothrombin time on or after POD5 based on the threshold values of the local laboratory (serum bilirubin: >1.2 mg/dl, prothrombin time: <75%). If a patient was suffering from an abnormal preoperative serum bilirubin level or prothrombin time, a postoperative aggravation on or after POD5 (compared to the previous day) was identified as postoperative LD. Of note, for patients who reached normal serum bilirubin or prothrombin time values prior to POD5 and were discharged early, due to good clinical performance, no further blood collection could be performed. Accordingly, these patients were considered as “no LD”.

Moreover, patients were followed for a period of 90 days and the occurrence of postoperative morbidity was prospectively recorded as previously described^8^. Furthermore, the severity of postoperative complications was classified in grade I to V according to the classification scheme published by Dindo *et al*.^22^. Briefly, in case of multiple complications per patient, the most serious one was graded. Accordingly, all patients requiring surgical, endoscopic or radiological intervention or were further suffering from life – threatening complications (including death), were further defined as “severe morbidity” (grade III–V).

### Statistical analyses

Statistical analyses were performed as previously described^8^. Briefly, statistical analyses were achieved using SPSS 20 software (SPSS, Inc., Chicago, IL, USA) and were based on non–parametric tests (Mann–Whitney U test, Wilcoxon test, chi–squared test). To further validate the diagnostic potential of ADAMTS13-AC to detect poor postoperative performance (i.e. LD, morbidity or severe morbidity) a “receiver operating characteristic” (ROC) curve analysis was performed. Additionally, this statistical approach was used to identify the optimal cut-off level to distinguish between high- and low- risk patients.

Boxplot illustrations are given without outliers and extreme values to improve the resolution of interquartile ranges. Values were defined as outliers if they exceeded 1.5 till 3 times of the interquartile range, while they were classified as extreme values if they exceeded more than 3 times of the inter quartile range. P values < 0.05 were considered statistically significant.

## Results

### ADAMTS13-AC deteriorates after liver resection in humans and is reflected by an increased VWF-AG/ADAMTS13-AC ratio

**Figure 1:**
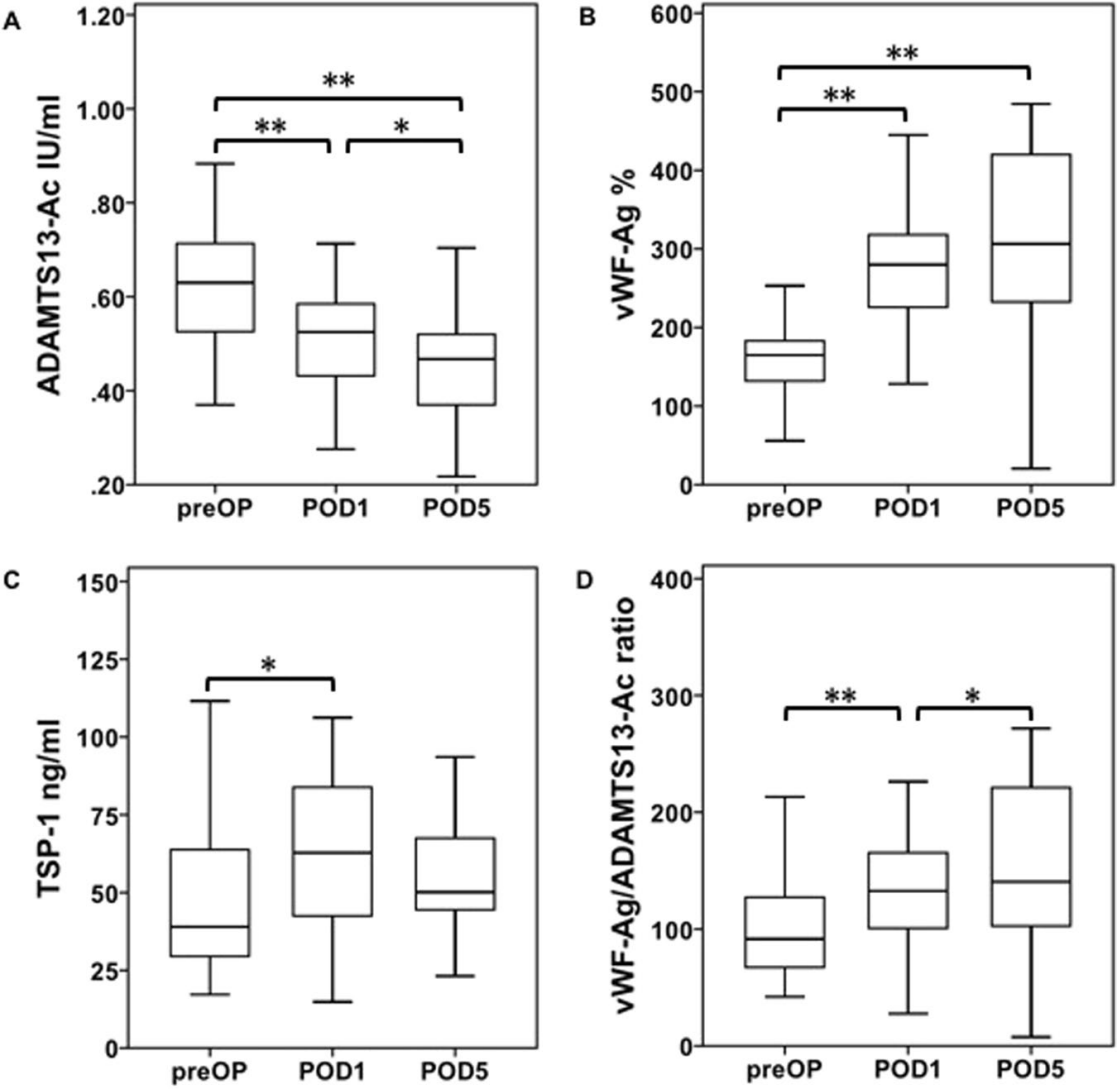
Perioperative time course of ADAMTS13-AC, VWF-AG, TSP-1 as well as VWF-AG/ADAMTS13-AC ratio. Perioperative activity values and antigen levels were measured one day prior to (PreOP), one day after (POD1) as well as 5 days after (POD5) liver resection and are shown separately in accordance to ADAMTS13-AC (**A**), vWF-Ag (**B**), TSP-1 (**C**) as well as vWF-Ag/ADAMTS13-AC ratio (**D**). Boxplot illustrations are given without outliers and extreme values to improve the resolution of interquartile ranges. ^*^P < 0.05, ^**^P < 0.005.

In order to characterize possible alterations of ADAMTS13-AC, we initially characterized the perioperative time course of ADAMTS13-AC in patients undergoing liver resection as illustrated in Fig. 1A. Interestingly, ADAMTS13-AC significantly decreased on POD1 (median: preOP = 0.6 IU/ml vs. POD1 = 0.5 IU/ml, p = 0.001), further diminished and remained decreased until POD5 (median: POD1 = 0.5 IU/ml vs. POD5 = 0.5 IU/ml, p = 0.01; median: preOP = 0.6 IU/ml, vs. POD5 = 0.5 IU/ml, p = 0.001).

Since ADAMTS13 is closely related to TSP-1 as well as vWF and its degradation into smaller less active fragments, we additionally characterized the perioperative time course of vWF-Ag (Fig. 1B) as well as TSP-1 (Fig. 1C). Accordingly, vWF-Ag significantly increased until POD1 (median: preOP = 164.9%, vs. POD1 = 280.0%, p = 0.001) and doubled till POD5 (preOP = 164.9% vs. POD5 = 306.3%, p = 0.001). This strong decrease of ADAMTS13-AC and increase of vWF-Ag on POD1 was accompanied with a concomitant increase of TSP-1 (median: preOP = 39.0 ng/ml, POD1 = 62.8 ng/ml, p = 0.02). Interestingly, TSP-1 tended to decrease up to POD5 but did not reach any significance when compared to preoperative values, or levels measured on POD1 (median: preOP = 39.0 ng/ml, vs. POD5 = 50.1 ng/ml, p = 0.70; median: POD1 = 62.8 ng/ml vs. POD5 = 50.1 ng/ml, p = 0.10).

The correlation of vWF-Ag and ADAMTS13-AC was reflected by a significant increase of vWF-Ag/ADAMTS13-AC ratio on POD1 (median: preOP = 268.9 vs. POD1 = 545.1, p = 0.001). The ratio further increased till POD5 (median: POD1 = 545.1 vs. POD5 = 751.9, p = 0.03, Fig. 1D) but failed to show any significant differences when compared to preoperative levels (median: preOP = 268.9 vs. POD5 = 751.9, p = 0.40).

### Perioperative ADAMTS13-AC levels are associated with poor postoperative outcome after liver resection

**Figure 2:**
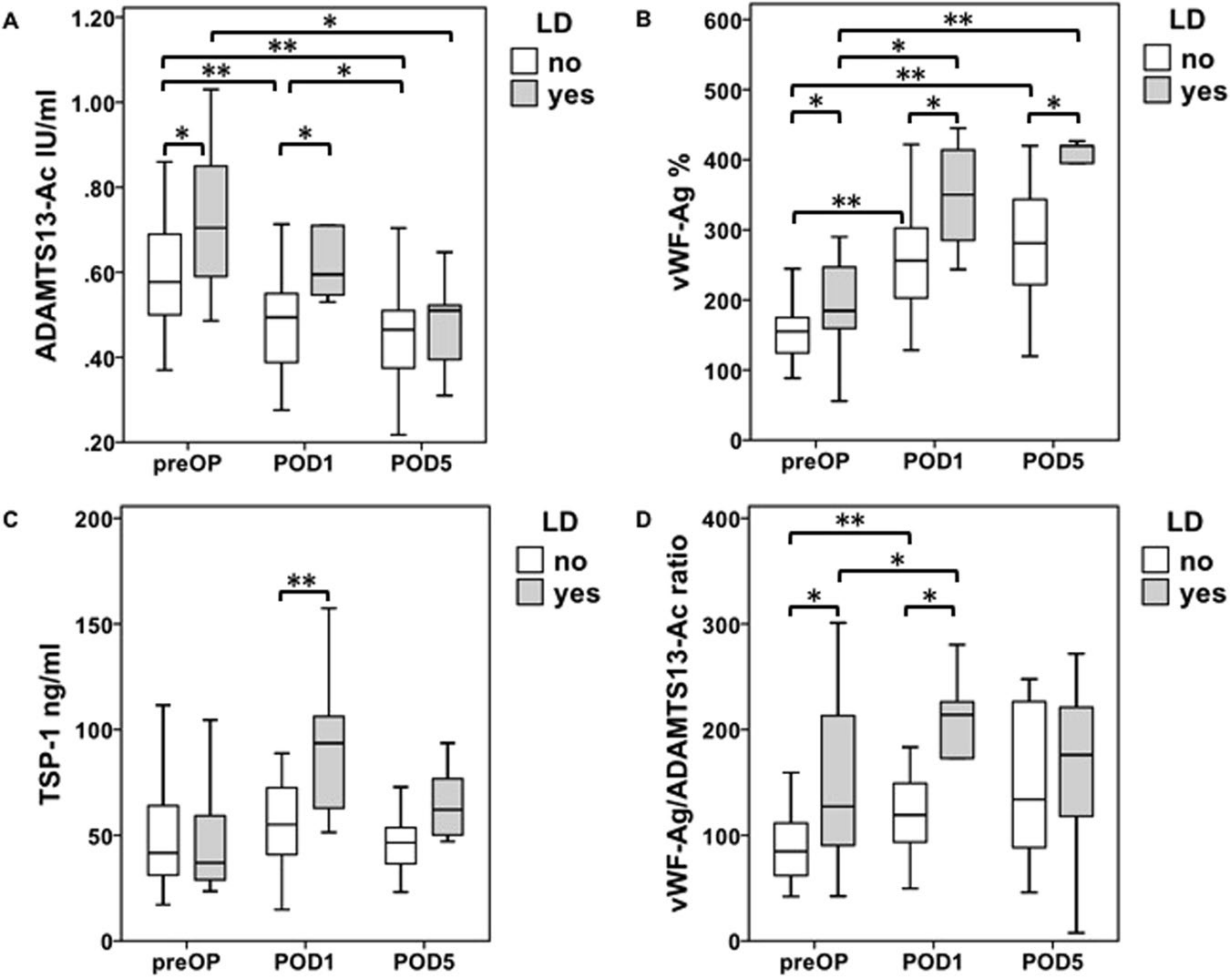
Perioperative time course of ADAMTS13-AC, VWF-AG, TSP-1 as well as VWF-AG/ADAMTS13-AC ratio in accordance to postoperative complications. Perioperative activity values and antigen levels were measured one day prior to (PreOP), one day after (POD1) as well as 5 days after (POD5) liver resection. Accordingly, ADAMTS13-AC (**A**), vWF-Ag (**B**), TSP-1 (**C**) as well as vWF-Ag/ADAMTS13-AC ratio (**D**) levels were evaluated and are shown in accordance to the occurrence of postoperative liver dysfunction (LD). Boxplot illustrations are given without outliers and extreme values to improve the resolution of interquartile ranges. ^*^P < 0.05, ^**^P < 0.005.

As the liver is known to play an essential role in hemostasis and hemostatic changes are thought to be one of the main inducers of LR after hepatectomy, we further evaluated possible associations between patients with poor and favorable postoperative development and perioperative ADAMTS13-AC levels. Of note, patients who lack a sufficient regenerative response suffered from significantly elevated preOP ADAMTS13-AC levels compared to those with normal hepatic recovery (preOP median: LD = 0.7 IU/ml vs. no LD = 0.6 IU/ml, p = 0.04, Fig. 2A). These findings persisted and were reflected by increased ADAMTS13-AC levels on POD1 in patients with postoperative LD compared to those with normal liver function (POD1 median: LD = 0.6 IU/ml vs. no LD = 0.5 IU/ml, p = 0.02, Fig. 2A). Interestingly, the observed diversity of ADAMTS13-AC levels between patients with and without LD could not be detected on POD5 (POD5 median: LD = 0.5 IU/ml vs. no LD = 0.5 IU/ml, p = 0.70, Fig. 2A). Moreover, in both patient groups, ADAMTS13-AC steadily decreased after hepatectomy, remained diminished and approximately equalized on POD5 (LD: preOP median = 0.7 IU/ml vs. POD5 = 0.50 IU/ml, p = 0.04; no LD preOP median = 0.6 IU/ml vs. POD5 = 0.5 IU/ml, p = 0.003, Fig. 2A).

As vWF and TSP-1 are closely related to ADAMTS13 we further evaluated vWF-Ag and TSP-1 dynamics in patients with and without postoperative LD. Accordingly, patients with poor postoperative regenerative response suffered from significantly increased preOP vWF-Ag levels compared to those with sufficient postoperative liver function (preOP median: LD = 184.5% vs. no LD = 155.0%, p = 0.048, Fig. 2B). This observation could also be observed on POD1 (POD1 median: LD = 350.2% vs. no LD = 256.0%, p = 0.02, Fig. 2B) and POD5 (POD 5 median: LD = 420.0% vs. no LD = 281.2%, p = 0.01, Fig. 2B). In addition to this, TSP-1 was significantly upregulated on POD1 in patients with postoperative LD (POD1 median: LD = 93.6 ng/ml vs. no LD = 55.1 ng/ml, p = 0.01, Fig. 2C). However preOP TSP-1 levels as well as TSP-1 values measured on POD5 failed to show any significant differences between patients with and without postoperative LD (preOP median: LD = 37.0 ng/ml vs. no LD = 41.7 ng/ml, p = 0.80; POD5 median: LD = 62.1 ng/ml vs. no LD = 46.6 ng/ml; p = 0.10, Fig. 2C).

To further evaluate the correlation between ADAMTS13-AC and vWF-Ag will translate into poor postoperative performance we additionally characterized the vWF-Ag/ADAMTS13-AC ratio of patients with poor and favorable postoperative hepatic recovery. Interestingly, patients with postoperative LD suffered from significantly elevated pre- as well as postoperative (POD1) vWF-Ag/ADAMTS13-AC ratio values, compared to patients without this complication (preOP median: LD = 127.3 vs. no LD = 84.8, p = 0.02; POD1 median: LD = vs. no LD=, p = 0.03, Fig. 2D).

### Preoperative ADAMTS13-AC predicts postoperative ld

**Figure 3:**
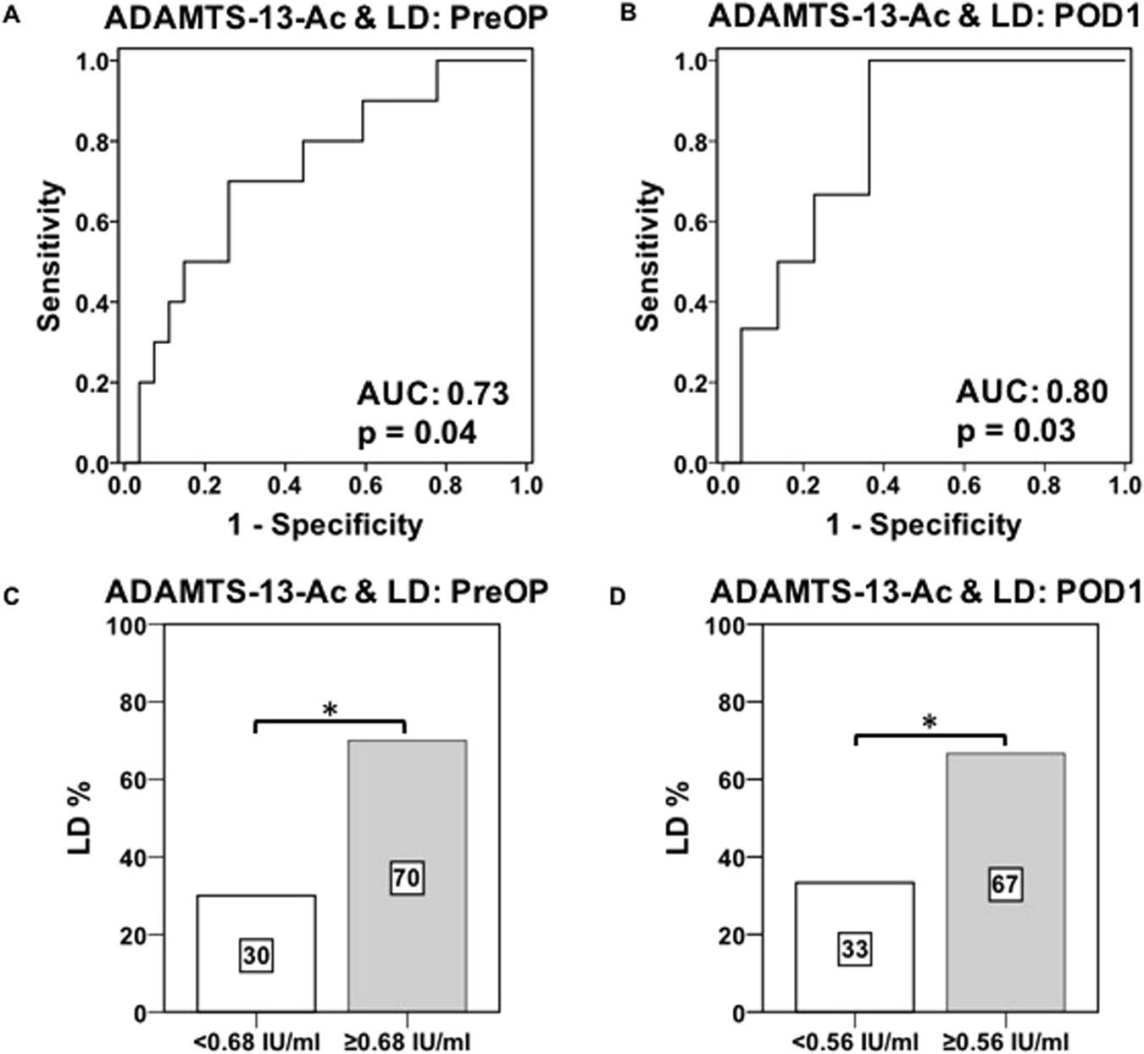
Pre- as well as postoperative potential of ADAMTS13-AC to predict postoperative LD. Receiver operating characteristic (ROC) curve analysis for preoperative (PreOP, **A**) as well as postoperative day 1 (POD1, **B**) ADAMTS13-AC values to detect postoperative liver dysfunction (LD) are illustrated. ROC curve analysis includes the evaluation of its related area under the curve (AUC). Furthermore incidences of postoperative LD are shown in accordance to preOP (≥0.68 IU/ml, **C**) as well as POD1 (≥0.56 IU/ml, **D**) defined cut-off values of ADAMTS13-AC. ^*^P < 0.05, ^**^P < 0.005.

As significant discrepancies in perioperative ADAMTS13-AC levels between patients with and without LD were observed, we further evaluated the predictive potential of ADAMTS13-AC to accurately detect patients who are at high risk of developing such severe complications. Therefore, ROC curve analysis was performed; revealing a significant predictive value of preOP ADAMTS13-AC levels (area under the curve [AUC]: 0.73, p = 0.04, Fig. 3A). Additionally, using ROC curve analysis, a preOP cut-off level of ADAMTS13-AC ≥ 0.68 IU/ml was chosen to precisely identify patients with postoperative LD, with a sensitivity of 70% and a specificity of 74%. To further evaluate whether unfavorable preOP ADAMTS13-AC levels would result in poor clinical performance, the incidence of postoperative LD was compared, using our preOP defined cut-off values of ADAMTS13-AC ≥ 0.68 IU/ml. Accordingly, patients fulfilling those preoperative cut-off levels suffered from significantly higher incidences of postoperative LD (ADAMTS13-AC < 0.68 IU/ml: 30% LD vs. ADAMTS13-AC ≥ 0.68 IU/ml: 70% LD, p = 0.01, Fig. 3C).

### Postoperative ADAMTS13-AC predicts postoperative ld

Since ADAMTS13-AC is exclusively produced within the liver and liver resection reduces functional liver mass we further aimed to assess if postoperative ADAMTS13-AC is able to differ between patients with and without postoperative LD. Therefore, to evaluate the predictive potential of postoperative measured ADAMTS13-AC, postoperative ROC curve analysis was performed. Accordingly, ROC curve analysis was able to accurately predict LD, revealing a significant predictive value of ADAMTS13-AC measured on POD1 (AUC: 0.80, p = 0.03, Fig. 3B). To additionally evaluate if postoperative elevated ADAMTS13-AC values would result in higher incidences of LD, we defined a postoperative cut-off of ADAMTS13-AC ≥ 0.56 IU/ml. Accordingly, patients fulfilling our postoperative cut-off values significantly suffered from higher incidences of LD (ADAMTS13-AC < 0.56 IU/ml: 33% LD vs. ADAMTS13-AC ≥ 56 IU/ml: 67% LD, p = 0.04; Fig. 3D), with a sensitivity of 67% and a specificity of 77%.

### VWF-AG to ADAMTS13-AC dysbalance reflects poor postoperative performance

**Figure 4:**
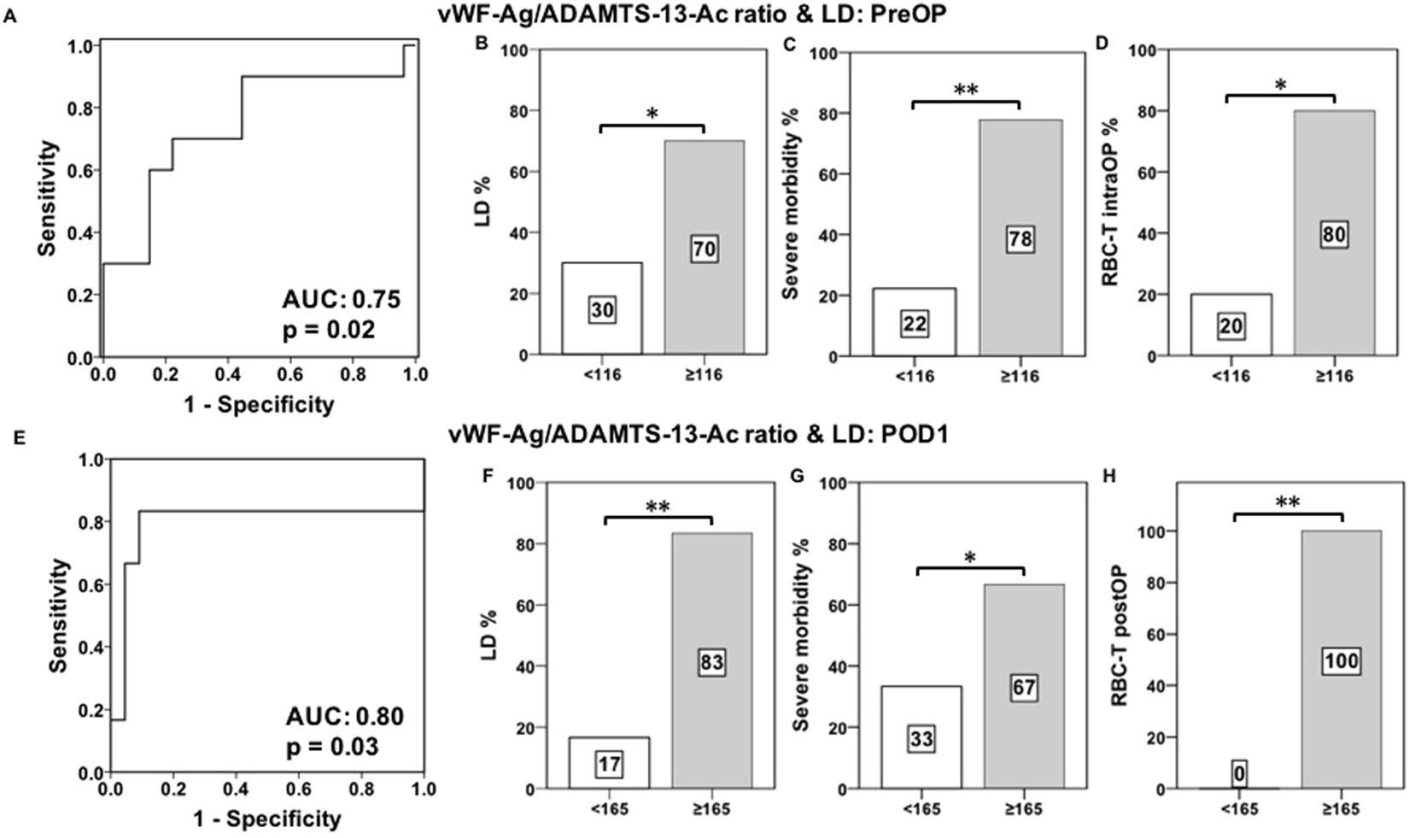
Perioperative potential of VWF-AG/ADAMTS13-AC ratio to predict poor postoperative performance. Receiver operating characteristic (ROC) curve analysis for preoperative (PreOP, **A**) as well as postoperative day 1 (POD1, **E**) vWF-Ag/ADAMTS13-AC ratio values to detect postoperative liver dysfunction (LD) are illustrated. ROC curve analysis includes the evaluation of its related area under the curve (AUC). Furthermore incidences of postoperative LD as well as severe morbidity and red-blood-cell transfusion (RBC-T) usage are shown in accordance to preOP (≥116, **B–D**) as well as POD1 (≥165, **F–H**) defined cut-off values of vWF-Ag/ADAMTS13-AC ratio. ^*^P < 0.05, ^**^P < 0.005.

As we had observed a correlation of ADAMTS13-AC and vWF-Ag, in patients with and without postoperative LD, during the perioperative course, we further aimed to evaluate the potential of vWF-Ag/ADAMTS13-AC ratio to predict poor postoperative performance. Accordingly a ROC curve analysis was performed revealing a significant predictive value of preoperative vWF-Ag/ADAMTS13-AC ratio (AUC: 0.75, p = 0.02; Fig. 4A) as well as postoperative vWF-Ag/ADAMTS13-AC ratio (AUC: 0.80, p = 0.03; Fig. 4E).

To see if vWF-Ag/ADAMTS13-AC ratio is able to distinguish between patients who are at higher risk of developing postoperative LD, further investigations were performed. Accordingly, based on our newly defined pre- as well as postoperative (POD1) cut-off levels of vWF-Ag/ADAMTS13-AC ratio (preOP: ≥116, POD1: ≥156) we were able to classify between patients who are at higher risk of developing postoperative complications. Patients fulfilling our pre- or postoperative cut-off values suffered from significantly higher incidences of postoperative LD (preOP: LD = 70% vs. no LD = 30%, p = 0.01, Fig. 4B; POD1: LD = 83% vs. no LD = 17%, p = 0.001, Fig. 4F) as well as postoperative severe morbidity (preOP: severe morbidity = 78% vs. no severe morbidity = 22%, p = 0.002, Fig. 4C; POD1: severe morbidity = 67% vs. no severe morbidity = 33%, p = 0.01, Fig. 4G). Moreover, a more pronounced dysbalance of vWF-Ag and ADAMTS13-AC was able to significantly predict intra- as well as postoperative usage of red-blood-cell transfusion (RBC-T). Accordingly, patients fulfilling our preOP vWF-Ag/ADAMTS13-AC ratio cut-off significantly suffered from higher incidences of intraoperative (intraOP) RBC-T (preOP: <116 = 20%, ≥116 = 80%, p = 0.02, Fig. 4D). Similar results could be observed in accordance to our postoperative vWF-Ag/ADAMTS13-AC ratio cut-off (POD1: <165 = 0%, ≥165 = 100%, p = 0.001, Fig. 4H).

## Discussion

The liver has the unique capacity to regenerate after multiple kinds of injuries^23^. Growing evidence suggests that platelets are one of the key players involved in a sufficient regenerative response after hepatectomy^8,9,24^. Hereof, vWF is known to be the main binding protein between activated endothelium and platelets and is therefore of crucial importance to support sufficient hemostasis and platelet granule release^25,26^. In this context, the vWF-cleaving protease ADAMTS13 has been shown to degrade vWF into less active fragments^27^. Recently, we were able to identify an essential role of vWF on site specific platelet accumulation and postoperative liver function recovery after liver resection in humans^11^. Applying a cut-off of vWF-Ag of 182% (preOP) and 315% (POD1) we were able to demonstrate that elevated vWF-Ag levels were vital to identify high-risk patients that suffered from an increased risk of poor postoperative outcome. Accordingly, same results could be observed when applying those cut-off values to this study cohort (≥182: LD = 60% vs. no LD = 40%, p = 0.01; ≥315: LD = 60% vs no LD = 40%, p = 0.01; suppl. Fig. 1).

With respect to this, a proper regulation of its activity seems to be essential to maintain the natural equilibrium of coagulability after certain kind of liver injuries^12,13,15^. However, up to now there is still a lack of evidence addressing the role as well as the predictive potential of ADAMTS13-AC in human LR after partial hepatectomy.

Within this study we were able to show that ADAMTS13-AC decreases after liver resection and that this decline is accompanied with a significant increase of vWF-Ag/ADAMTS13-AC ratio. Additionally, ADAMTS13-AC was significantly elevated in patients that developed postoperative LD. This elevation of ADAMTS13-AC in patients with diminished postoperative regenerative response was paralleled by significantly higher postoperative vWF-Ag levels. Further, we documented ADAMTS13-AC to be a valuable marker to predict postoperative LD after partial hepatectomy. Importantly, with reference to the central relevance of vWF in hemostasis, patients with deteriorated pre- or postoperative vWF-Ag/ADAMTS13-AC ratio levels also suffered from significantly higher incidences of postoperative LD as well as perioperative usage of RBC-T.

ADAMTS13 production is thought to be exclusively related to liver-specific stellate-cells and its activity has been shown to decrease due to chronic or acute liver injury and thereby loss of functional liver mass^12,28^. Due to its proteolytic activity, ADAMTS13 has the potential to cleave vWF and further degrade it into less active fragments^16^. However, as a reflection of liver damage and endothelial cell activation vWF-Ag is secreted from its main storage, the Weibel-Palade bodies, and increases after chronic or acute liver injuries^12,26^. Accordingly, we recently demonstrated that vWF-Ag is of crucial importance to mediate platelet aggregation and thereby improve postoperative LR^11^. Indeed, within this study we were able to show similar results. ADAMTS13-AC deteriorated after hepatectomy, being accompanied by an up regulation of vWF-Ag up to five days after surgery. This intraoperative dynamics culminated in an increased postoperative vWF-Ag/ADAMTS13-AC ratio and was further associated with increased TSP1 levels on POD1.

Reduced hepatic regeneration was associated with significantly elevated perioperative ADAMTS13-AC levels compared to patients with a sufficient regenerative response after hepatectomy. Although the precise mode of ADAMTS13 production has not yet been identified, recent studies suggest hepatic stellate cells (HSC) to be one of the main sources of ADAMTS13 secretion upon their activation^28^. Accordingly, several different pathways of HSC activation have been proposed^29^. On the basis of liver injury, two major mechanisms can be separated^29^: 1) In case of liver injury, HSC differentiate into activated myofibroblast-like cells and secret their contents to protect the liver from further damage^30,31^. 2) Chronic tissue damage will lead to recruitment of inflammatory cells as well as liver sinusoidal cells and in doing so promote HSC activation and fibrosis^31^. In this context, ADAMTS13 was shown to correlate with severity of fibrosis and its activity was impaired in patients with mild to moderate fibrosis, as a sign of consecutive activation and secretion of ADAMTS13 proteinase. Nevertheless, due to the constant stimulus and thereby exhaustion of HSC, ADAMTS13-AC was significantly reduced in patients with severe fibrosis/cirrhosis^32^.

Interestingly, within this study no significant correlation between the grade of fibrosis and perioperative vWF-Ag as well as TSP-1 levels or development of postoperative LD could be detected. With respect to fibrosis grading and only when measured on POD1 and POD5, ADAMTS13-AC was irregularly distributed. However, since the number of patients is limited with respect to each of the 4 groups (2, 11, 6, 5 and 8 patients), the power to detect those suggested differences might be too low (suppl. Table 1).

In this context and as a sign of pre-damaged liver tissue, patients with postoperative LD showed significantly higher perioperative values of ADAMTS13-AC.

Interestingly, patients with postoperative LD showed not only elevated ADAMTS13-AC levels but also significantly increased vWF-Ag levels. Accordingly, liver sinusoidal endothelial cells (LSEC) are well known to get activated and undergo phenotypical changes due to ongoing and chronic liver injury^33^. As a consequence of their activation, LSECs secret their contents and thus interact with their environment via paracrine signals. Hereof, vWF is known to be secreted upon endothelial cell activation, and have recently been found to correlate with the degree of fibrosis and presence of portal hypertension^7,34^. In this context, as a reflection of chronic liver injury and fibrogenesis, LSECs seems to be constantly stimulated to secret their contents and thereby leading to elevated baseline levels of vWF-Ag in patients with postoperative LD. Furthermore, our data suggest a relative link between elevated levels of ADAMTS13-AC and vWF-Ag in patients with postoperative LD. Deleve *et al*. revealed that under normal conditions, LSECs suppresses the activation of HSC, but following injury, LSECs lose this capacity and further promote HSC activation^33^. As a consequence, ongoing liver injury and fibrosis leads not only to elevated vWF-Ag levels but also to increased ADAMTS13-AC.

Furthermore, ADAMTS13 is known to significantly influence postoperative hemostasis and is thereby able to cause either thrombotic or hemorrhagic effects^15^. Recent studies suggest a link between increased ADAMTS13-AC levels and disrupted coagulation. Given the fact that hemostasis is of central relevance during liver surgery, substantially increased ADAMTS13-AC levels should lead to a reduced amount of vWF-multimers and thereby accelerate perioperative blood loss^16,35^. In line with these results, we found vWF-Ag/ADAMTS13-AC ratio to be able to significantly predict perioperative RBC-T usage. This is of further clinical relevance; as previous studies have revealed that perioperative usage of blood transfusion is associated with worse clinical outcome (i.e. increased risk of morbidity, mortality and long term survival) after oncologic hepatectomy.

Up to now, postoperative risk stratification still endures to be a challenging assignment in liver surgery. Following resection, the liver has to enhance liver mass revitalization while maintaining its vital and organ specific function. A collapse of this physiological course will result in one of the most severe complications following hepatectomy, namely postoperative LD. In the past, several studies have addressed the relevance of ADAMTS13 and its activity during perioperative time-course after liver surgery^12,13,35,36^. Nevertheless, most of them lack sufficient evaluation of ADAMTS13-AC and its predictive potential to detect patients who might be affected by post-hepatectomy liver failure. Accordingly, within this study we were able to show that preoperative ADAMTS13-AC measurement on POD1 were able to accurately identify high-risk patients who require a more intense surveillance and therefore might represent a useful tool for perioperative risk stratification in patients undergoing hepatic resection. In particular, patients who fulfilled our pre- as well as postoperative defined cut-off values of ADAMTS13-AC as well as vWF-Ag/ADAMTS13-AC ratio, suffered from significantly higher incidences of postoperative LD. Moreover, one patient died within our cohort. Importantly, this patient did fall in our high-risk group in terms of our pre- and postoperative cut off (Table 1 and Table 2). Accordingly, vWF-Ag/ADAMTS13-AC “high” patients might benefit from detailed monitoring to detect postoperative liver failure. Furthermore, the timely initiation of liver support devices might be guided by perioperative ADAMTS13-AC measurement.

During the last 2 decades various methods have been developed to accurately determine ADAMTS13-activity^37,38^. In general, two groups of detection-method can be separated but both of them are based on the determination of residual undigested vWF-substrate. Nevertheless, there are some major differences when it comes down to clinical applicability^37,38^. One of the detection methods is based on multimeric vWF and is postulated to be reproducible but in contrast less sensitive than the other testing system and furthermore often requiring equipment, which is uncommon in routine diagnostic laboratories. Moreover, a major disadvantage of this method is that it requires the usage of denaturing agents, which are thought to modify the binding modalities of free ADAMTS13-Ag and bound autoantibodies and therefore prolong the assay reaction time (6–24 h)^37,38^. On the other hand, the second method is based on vWF-peptides. It is also reproducible, more sensitive than the primarily established determination method, but not requiring denaturing agents which dramatically shortens the reaction time down to 0, 5–3 hours. Based on those second method several assays have been established which further reduces the complexity of ADAMTS13-AC detection and simplifies the techniques and instruments down to standard ELISA equipment^37,38^. Ultimately, this points out that our results will have to be validated in a large prospective setting including multiple routine laboratories of large hepatobiliary centers to determine comparability of ADAMTS-13 measurement and establish a relevant cut-off for clinical use.

Moreover, some relevant weaknesses of this study should be acknowledged. In particular, the evaluated number of patients is fairly small. However, due to meticulous sample preparation, we were able to document specific differences between patients with poor clinical performance and perioperative ADAMTS13 values, despite the limited sample size. Moreover, it should be noted that not all patient could be followed up and analyzed during the perioperative time course. In particular, there is a lack of data from 9 patients on POD1, from which no plasma could be collected. Accordingly, this might affect postoperative ADAMTS13 results due to the reduction in power and should be taken into consideration during result interpretation. In this context, there seems to be a correlation between the extent of resection and postoperative LD. However, those differences failed to become significant and did not affect perioperative vWF-Ag/ADAMTS13-AC ratio values (Table 1 and Table 2). Ultimately, these results should be validated in a larger prospective setting.

In conclusion, ADAMTS13-AC deteriorated after liver resection in humans and was associated with increased levels of vWF-Ag. Patients with postoperative LD suffered from increased levels of ADAMTS13-AC as well as significantly up regulated vWF-Ag levels. Further, we revealed ADAMTS13-AC as well as vWF-Ag/ADAMTS13-AC ratio to be valuable markers to predict postoperative LD after partial hepatectomy. Patients with deteriorated pre- or postoperative ADAMTS13-AC levels suffered from significantly higher incidences of LD. Therefore; perioperative ADAMTS13-AC measurement might represent a useful tool to distinguish between patients who are at higher risk of developing such severe postoperative complications. Indeed, further investigations have to be performed to consolidate its role as a predictive marker to detect postoperative LD.

## Electronic supplementary material

Supplementary Information

## Data Availability

All data generated or analyzed during this study are included in this published article (and its supplementary information files).
