# Supplementary material for: Elevated ADAMTS13 Activity is Associated with Poor Postoperative Outcome in Patients Undergoing Liver Resection
Source: Sci Rep. 2018 Nov 14;8:16823. doi: 10.1038/s41598-018-34794-w (PMC6235878; doi:10.1038/s41598-018-34794-w)
Supplement: Supplementary file 1 — Supplementary Information [file 41598_2018_34794_MOESM1_ESM.pdf]

Elevated ADAMTS13 Activity is Associated with Poor Postoperative Outcome in Patients Undergoing Liver Resection

Stefanie Haegele[1], Jennifer Fuxsteiner[1], David Pereyra[1], Christoph Koeditz[1], Benedikt Rumpf[1], Clara Schütz[1], Christian Schwarz[1], Christine Brostjan[1], Thomas Gruenberger[2], Patrick Starlinger[1]

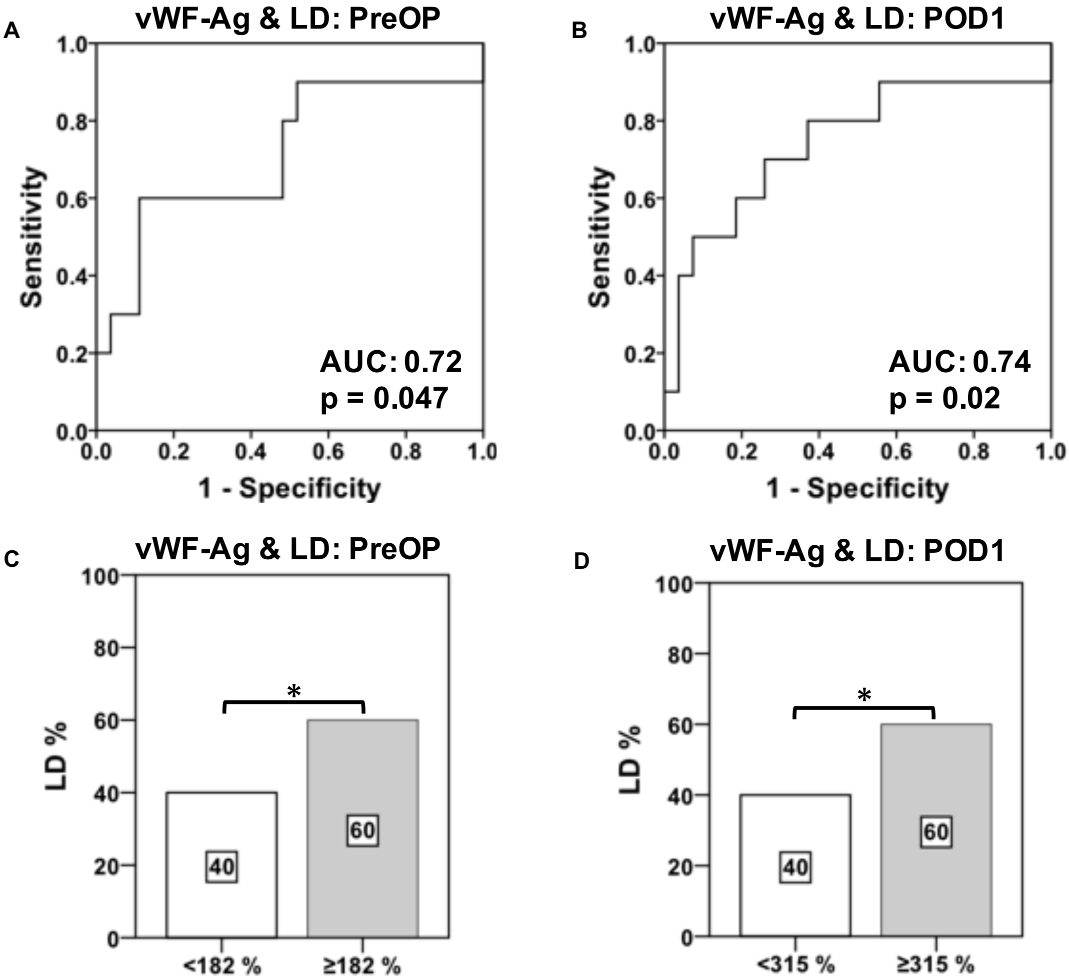

SUPPLEMENTARY FIGURE 1, PRE- AS WELL AS POSTOPERATIVE POTENTIAL OF VWF-AG TO PREDICT POSTOPERATIVE LD

Receiver operating characteristic (ROC) curve analysis for preoperative (PreOP, A) as well as postoperative day 1 (POD1, B) vWF-Ag values to detect postoperative liver dysfunction (LD) are illustrated. ROC curve analysis includes the evaluation of its related area under the curve (AUC). Furthermore incidences of postoperative LD are shown in accordance to preOP (≥ 182%, C) as well as POD1 (≥ 315%, D) defined cut-off values of vWF-Ag.
* P <0.05, * * P <0.005.

**Supplementary table 1; Perioperative Levels of LD, Fibrosis, vWF-Ag, ADAMTS13-Ac and its ratio**							
**Parameter**		**Fibrosis grade** (FG, N = 32), *5 cases are missing*					
		**FG 0** (N = 2)	**FG 1** (N = 11)	**FG 2** (N = 6)	**FG 3** (N = 5)	**FG 4** (N = 8)	**P - value**
		**N=/ Median (Range/ %)**					
**ADAMTS13-ac IU/ml**							
preOP		0.68 (0.64 – 0.72)	0.63 (0.39 – 0.38)	0.55 (0.37 – 0.88)	0.56 (0.46 – 0.85)	0.69 (0.53 – 1.11)	0.12
POD1		0.42 (0.42 – 0.42)	0.45 (0.22 – 0.51)	0.45 (0.34 – 0.58)	0.55 (0.37 – 0.71)	0.63 (0.51 – 0.66)	**0.04**
POD5		0.31 (0.31 – 0.31)	0.44 (0.22 – 0.51)	0.52 (0.52 – 0.52)	0.43 (0.38 – 0.47)	0.52 (0.42 – 0.70)	**0.006**
**vWF-Ag %**							
preOP		298 (175 – 420)	153 (56 – 178)	165 (124 – 290)	160 (125 – 245)	180 (99 – 253)	0.10
POD1		338 (256 – 420)	215 (49 – 384)	233 (171 – 414)	313 (249 – 329)	311 (190 – 445)	0.86
POD5		420 (420 – 420)	177 (21 – 395)	300 (236 – 427)	360 (299 – 484)	420 (313 – 420)	0.41
**TSP-1 ng/dl**							
preOP		41.4 (35.2 – 47.6)	47.3 (20.8 – 179.4)	44.6 (17.3 – 59.3)	118.0 (30.1 – 195.9)	30.9 (17.2 – 64.3)	0.61
POD1		91.5 (36.3 – 146.7)	68.9 (15.2 – 106.2)	54.5 (14.9 – 157.4)	55.1 (34.0 – 303.0)	77.7 (38.2 – 242.0)	0.53
POD5		93.6 (93.6 – 93.6)	45.0 (25.8 – 72.8)	48.6 (23.2 – 53.6)	49.3 (36.5 – 62.1)	62.4 (43.9 – 82.4)	0.24
**vWF-Ag/ADAMTS13-ac ratio**							
preOP		206.2 (111.4 – 301.1)	87.6 (42.0 – 141.8)	83.5 (48.8 – 256.1)	98.1 (57.5 – 193.3)	126.8 (67.2 – 213.2)	0.23
POD1		107.9 (107.9 – 107.9)	117.5 (27.7 – 203.7)	108.0 (58.1 – 226.2)	137.0 (115.8 – 224.4)	267.3 (138.4 – 280.4)	0.15
POD5		130.2 (130.2 – 130.2)	61.6 (7.6 – 105.6)	219.7 (219.7 – 219.7)	133.9 (127.3 – 140.5)	222.6 (176.0 – 247.8)	**0.03**
**vWF-AG/ ADAMTS13-ac ratio**							
preOP *5 cases are missing*	<116	1 (50.0)	9 (81.8)	4 (66.7)	3 (60.0)	**1** (33.3)	0.40
	≥116	1 (50.0)	2 (18.2)	2 (33.3)	2 (40.0)	**2** (66.7)	
POD1 *1 case is missing*	<165	1 (50.0)	9 (81.8)	**4** (80.0)	3 (60.0)	3 (37.5)	0.45
	≥165	1 (50.0)	2 (18.2)	**1** (20.0)	2 (40.0)	5 (62.5)	
**LD**							
yes		1 (50.0)	3 (27.3)	2 (33.3)	1 (20.0)	2 (25.0)	0.94

ADAMTS13- ac, metalloproteinase with thrombospondin type 1 motive activity; FG, fibrosis grade; LD, liver dysfunction; POD, postoperative day; preOP, preoperative; vWF-Ag, von Willebrand factor antigen;
